# Self-Assembled Structures of Colloidal Dimers and Disks on a Spherical Surface

**DOI:** 10.3390/e23050585

**Published:** 2021-05-09

**Authors:** Nkosinathi Dlamini, Santi Prestipino, Giuseppe Pellicane

**Affiliations:** 1 School of Chemistry and Physics, University of Kwazulu-Natal and National Institute of Theoretical Physics (NIThEP), Pietermaritzburg 3209, South Africa; Dlamini2@ukzn.ac.za; 2 Dipartimento di Scienze Matematiche ed Informatiche, Scienze Fisiche e Scienze della Terra, Università degli Studi di Messina, Viale F. Stagno d’Alcontres 31, 98166 Messina, Italy; sprestipino@unime.it; 3 Dipartimento di Scienze Biomediche, Odontoiatriche e delle Immagini Morfologiche e Funzionali, Università degli Studi di Messina, 98125 Messina, Italy; 4 CNR-IPCF, Viale F. Stagno d’Alcontres, 98158 Messina, Italy

**Keywords:** molecular self-assembly, amphiphilic aggregates, spherical boundary conditions

## Abstract

We study self-assembly on a spherical surface of a model for a binary mixture of amphiphilic dimers in the presence of guest particles via Monte Carlo (MC) computer simulation. All particles had a hard core, but one monomer of the dimer also interacted with the guest particle by means of a short-range attractive potential. We observed the formation of aggregates of various shapes as a function of the composition of the mixture and of the size of guest particles. Our MC simulations are a further step towards a microscopic understanding of experiments on colloidal aggregation over curved surfaces, such as oil droplets.

## 1. Introduction

Colloidal particles dispersed in a fluid medium are widely considered to be an ideal system where self-assembly can be explored, since they can be resolved and tracked in real time using optical microscopy [1]. The aggregation of colloidal particles is often the outcome of steric stabilization by electrostatic repulsion, which is achieved by modifying the salt concentration or by adding chemicals as stabilizing agents, as in the case of gold colloids [2] or silica and polystyrene particles [3,4]. The morphology of colloidal aggregates depends on the prevailing aggregation mechanisms and on particle shape [1], and is typically observed in ramified or compact clusters of fractal dimensionality [5], in a number of crystalline and amorphous solids [6], and in mesophases [7,8,9], which are partially ordered phases that are intermediate between liquids and crystals (e.g., cluster fluids, liquid crystals, and quasicrystals). Colloidal nanocrystals are even able to self-assemble in crystalline superlattices with an intricate structure [10].

In the last few decades, many researchers focused on the self-assembly of colloidal particles at an interface, which may serve as a scaffold or template for particle aggregation. Assembly at air–liquid and liquid–liquid interfaces is driven by a complex interplay of entropic and enthalpic forces [11]. The ability of oil–water interfaces to trap micron-sized particles has been known for over a century [12,13], and the strong binding of colloidal particles to fluid interfaces (the binding energy is even thousand times stronger than the thermal energy) is also evidenced by the stabilization of foams and emulsions against decomposition [14,15]. The self-assembly of colloidal particles on a flat surface can only rely on the control of interparticle interactions at the interface [16,17,18,19,20]. However, thanks to recent progress in microfluidics, it is possible to modify the interfacial geometry to trap colloidal particles, thus extending the initial range of applications of colloidal self-assembly. Indeed, curved phases of matter are found in a large number of systems, including biological entities such as cells and viral capsids, and the competition between the tendency to self-assemble and the geometric frustration originated by the curved interface can lead to novel structures, which are simply impossible to obtain over flat interfaces [21]). Recently, spherical boundary conditions have begun to also be employed in the realm of ultracold quantum particles, where “phases” with polyhedral symmetry are expected [22], and Bose–Einstein condensation has peculiarities that are experimentally well within reach [23].

Concrete realizations of spherical crystals are found in the emulsions of two immiscible fluids, such as oil and water, which are stabilized against droplet coalescence by coating the interface of one of the fluids with small colloidal particles [24]. A nontrivial issue is overcoming the strong binding of colloidal particles to the (liquid–liquid) interface and allowing them to diffuse quickly enough, i.e., like in a true fluid, to facilitate self-assembly over the substrate. Recently, that was achieved by very efficient functionalization with complementary DNA strands of both the surface of oil droplets (which was stabilized with sodium dodecyl sulphate (SDS), i.e., a micelle-forming surfactant) and the surface of colloidal particles [25]. Fluidlike diffusion was reached by allowing colloidal particles to anchor on rafts of polylysine-g[3.5]-polyethylene glycol-biotin (PLL-PEG-bio), which are free to slide on the surfactant. Upon increasing the concentration of SDS, the colloidal particles attached to the surface were observed to undergo aggregation as a result of the depletion effect driven by the excluded area of the surfactant micelles [25].

Spherical droplets were also coated with polystyrene latex particles [26] to form aggregates with a rigid shell, called colloidosomes in analogy to liposomes [27]. Structures resulting from the encapsulation of colloidal particles [28] are potential candidates for the delivery of drugs and vaccines, and may be used as vehicles for the slow release of cosmetic and food supplements.

In general, the self-assembly of colloidal particles on a spherical surface is an important paradigm to understand the structuring of membrane cells, which exhibit stable domains. The distribution and composition of these domains over the cell surface determines the interaction energy between different cells, ultimately driving the organization of the crowded environment inside biological organisms, where a huge number of cells is present [29].

Recently, we studied in 3D space [30] and on a plane [31] an implicit-solvent description of the dispersion of two colloidal species, namely, an amphiphilic dimer and a guest spherical/circular particle, where the smaller monomer in the dimer was solvophobic and had a strong affinity for the guest particle. In this paper, we consider the same system embedded in a spherical surface. By establishing bonds with two nearby curved disks, the smaller monomer provides the glue that keeps the disks together, which is the mechanism by which disks can form aggregates. However, once an aggregate of disks is covered with dimers, further growth of the aggregate is obstructed by the steric hindrance of the coating shell. Since the dimer–disk attraction is of limited range, mostly zero (“micelles”)- and one-dimensional aggregates (“chains”) are expected to form for a moderately low number of disks, while two-dimensional self-assembled structures (i.e., stratified lamellae) could occur under equimolar conditions.

The paper is organized as follows. In Section 2, we describe the model and employed method. In Section 3, we present and discuss our results. Lastly, we report our conclusions in Section 4.

## 2. Model and Method

The investigated model is the curved-surface analog of the same mixture of dimers and spherical guest particles that was studied in [30,31,32]. A dimer consists of two tangent hard calottes (i.e., disks following the surface of the sphere) with curved diameters $\sigma_{1}$ and $\sigma_{2}=3\sigma_{1}$, whereas guest particles are represented as hard calottes of size $\sigma_{3}$ (below, we generically refer to these particles as “disks”). In addition to the impenetrability of all particle cores, we added an attraction between the small monomer and the disk, modeled as a square-well potential of depth ϵ; the width of the well was set to be equal to $\sigma_{1}$. In the following, $\sigma_{2}$ (i.e., the diameter of the large monomer) and ϵ are taken as units of length and energy. Lastly, $N_{1}=N_{2}$ and $N_{3}$ are the number of dimers and disks, respectively; hence, $N=N_{1}+N_{3}$ is the total number of particles and $\chi =N_{3}/N$ is the (disk) composition.

Most data were collected for a fixed number $N_{3}=400$ of disks with diameter $\sigma_{3}=\sigma_{2}$ and varying composition. Number density was $\rho^{*}\equiv \left(N/A\right)\sigma_{2}^{2}=0.05$ (with *A* being the area of the spherical surface), but we also performed a few simulations for $\rho^{*}=0.25$ to probe the regime of moderately high curvatures. We analyzed the system behavior for a number of compositions: χ=20%,33%,50%, and 80%. Once *N* and χ are set, the number of dimers follows accordingly (sphere radius *R* is uniquely determined from *N* and $\rho^{*}$). We also examined how self-assembly changes when the disk diameter is increased up to $5\sigma_{2}$.

Simulations were carried out using the standard Metropolis algorithm in the canonical ensemble. Typically, a few hundred million Monte Carlo (MC) cycles are performed, one cycle consisting of *N* trial moves. Both translational and rotational moves are carried out for dimers. In performing a translational move, the midpoint of the arc joining the monomer centers is randomly shifted on the sphere, while keeping the direction of the subtended chord fixed in the embedding three-dimensional space. In a rotational move, it is the midpoint of the arc between the monomers that is fixed, while the chord is rotated at random. The maximal random shift and rotation were adjusted during the equilibration run, so as to keep the ratio of accepted to total number of moves close to 50%. The schedule of each move was designed so that the detailed balance held exactly. Particles are initially distributed at random on the sphere (using a variant of the Box–Muller algorithm [33]). Then, the system is quenched to $T^{*}\equiv k_{B}T=0.15$ or 0.10 and subsequently relaxed until some stationary condition is established. We checked that a slow cooling of the system, starting at each temperature from the last configuration produced at a slightly higher temperature, did not make any substantial difference in the structural properties of the steady state, because simulated systems are overall dilute.

**Figure 1:**
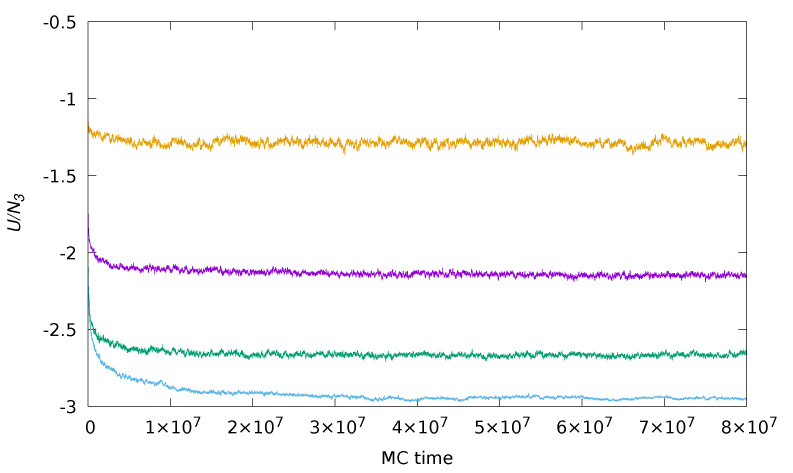
Energy evolution as a function of Monte Carlo time for $\sigma_{3}=\sigma_{2},T^{*}=0.10,\rho^{*}=0.05$, and various disk compositions (from top to bottom, χ=20%,33%,50%, and 80%).

A property signalling how far the system is away from equilibrium is the total potential energy *U*: an energy fluctuating around a fixed value for long is the hallmark of (meta)stable equilibrium. In ϵ units, *U* gives the total number of 1–3 contacts in the current system configuration. Hence, a stationary value of *U* indicates that aggregates eventually reached a nearly stable structure. Typically, $10^{8}$ cycles suffice for reaching a stationary state of low density. This is clearly illustrated in Figure 1, showing the energy evolution as a function of Monte Carlo cycles for $T^{*}=0.10,\rho^{*}=0.05$, and a number of compositions. Once equilibrium (or whichever steady state) is reasonably attained, we gain insight into the nature of aggregates mainly by visual inspection. We also computed the radial distribution function (RDF) of disks, $g_{33}\left(r\right)$ in a rather long production run of $10^{7}$ cycles (we checked that statistical errors on the RDFs were indeed negligible). For the sake of comparison, similar studies of 2D binary mixtures at considerably higher total density were executed for one order of magnitude fewer MC steps [34]. Even in a strongly heterogeneous system where mesoscopic structures were present, $g_{33}\left(r\right)$ bears valuable information on the arrangement of disks in the close neighborhood of a reference disk. Two disks form a bound pair when their distance is not larger than $r_{\min}=\sigma_{3}+3\sigma_{1}$ [31]: this is the maximal distance at which two disks can still be in contact with the same small monomer (exactly placed in the middle). For $\sigma_{3}=\sigma_{2}$, this implies that a disk forms bonds with all its first and second neighbors, identified as such through the RDF profile.

## 3. Results

We first comment on the simulation results for a mixture of disks and large monomers having the same size (Section 3.1). We consider systems of both low density ($\rho^{*}=0.05$) and moderate density ($\rho^{*}=0.25$). Next, we examine what changes when dimers are much smaller than the disks (Section 3.2). By visual inspection, we could easily ascertain the nature of the structures present in the stationary configurations of the low-temperature system.

### 3.1. Same Size of Dimers and Disks

**Figure 2:**
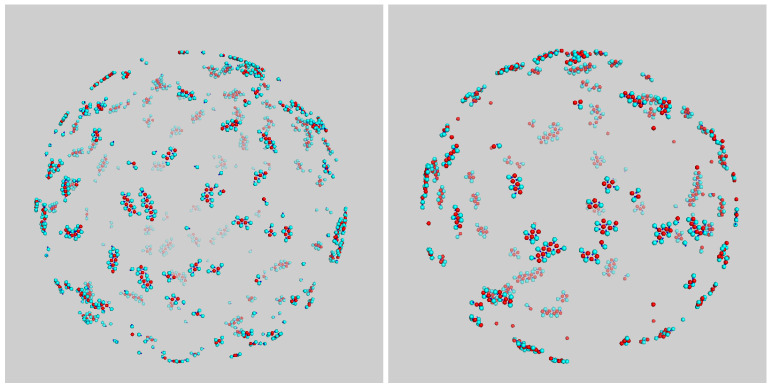
Typical configuration of mixture for $\sigma_{3}=\sigma_{2}$ and $\rho^{*}=0.05$ after a long run at $T^{*}=0.15$. Snapshots refer to a system of composition (**left**) χ=33% and (**right**) χ=50%.

We initially set the density to be equal to $\rho^{*}=0.05$, and disk size to $\sigma_{3}=\sigma_{2}$. For $T^{*}=0.15$, the equilibrated system is a fluid of small globular clusters for all disk compositions; see examples in Figure 2. Only for values of χ lower than about 10% did dimers form a well-definite coating shell around disks. Thermal fluctuations for $T^{*}=0.15$ are still too important to allow for the formation of more elaborate structures.

**Figure 3:**
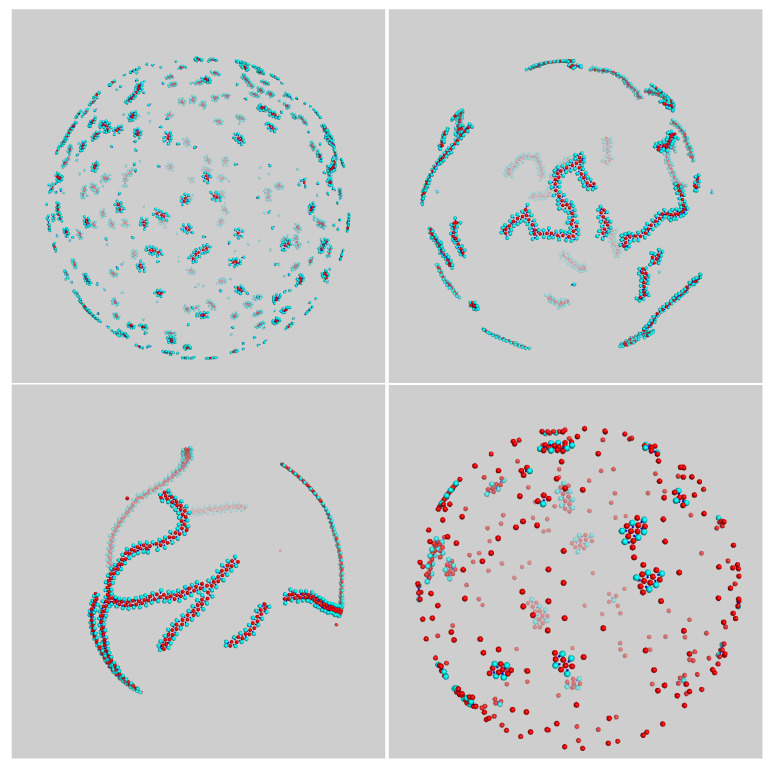
Typical configuration of mixture for $\sigma_{3}=\sigma_{2}$ and $\rho^{*}=0.05$ after a long run at $T^{*}=0.10$. Snapshots were taken at different compositions: (from **top left** to **bottom right**) χ=20%,33%,50%, and χ=80%.

Things changed radically for $T^{*}=0.10$, where the nature of aggregates was more varied. For χ=20% or lower, we invariably observed small groups of disks surrounded by dimers (Figure 3, top-left panel); for higher compositions up to 50%, aggregates were more elongated and wormlike (see top-right and bottom-left in Figure 3). A closer look at such “worms”, which are obviously the 2D analog of lamellae, revealed that they were assembled from a repeating unit, like a polymer chain. For still higher χ, the mean size of aggregates returned to being small again, since the number of gluing dimers was insufficient for all disks, and many disks then remained unbound (Figure 3, bottom-right panel). Therefore, aggregates only achieved large sizes when the number of disks roughly matched that of dimers.

The dynamics of aggregation in the present model is easy to explain. Initially, when the system was still disordered, the aggregation of disks proceeded very fast through the formation of bonds between disks and dimers. As an aggregate grows in size, however, its surface becomes increasingly rich in large monomers, which are inert particles; eventually, an aggregate stops growing when its disks and small monomers all lie buried under the surface. While local adjustments of the structure still occur at a high rate, the merging of two disconnected aggregates (or the breaking of a long chain) is highly suppressed and only takes place on much longer time scales. The existence of two regimes of aggregate growth (fast and slow), corresponding to a transition from diffusion-limited to reaction-limited aggregation [3], is reflected in the crossover of *U* from an exponential to a subexponential decay (as evidenced in Figure 1).

For $T^{*}=0.10$, the mechanism underlying the structure of aggregates is mainly energy minimization, whereas entropy considerations play a minor role. However, entropy is decisive in shaping the large-scale distribution of aggregates on the sphere, inasmuch as their structure maintains a certain flexibility (see more below). The relative size of particles and the range of 1–3 attraction are also clearly important. It is the short-range character of the attraction that is responsible for the essentially one-dimensional geometry of the larger aggregates.

**Figure 4:**
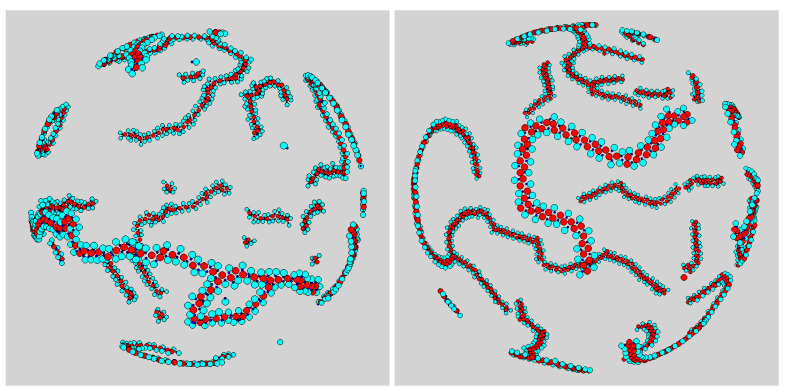
Typical configurations of mixture for $\sigma_{3}=\sigma_{2}$ after “only” $3\times 10^{7}$ MC cycles. Snapshots were taken for $T^{*}=0.10$ and $\rho^{*}=0.05$, and refer to (**left**) χ=33% and (**right**) χ=50%.

Particularly interesting are the systems with χ=33% and χ=50%, which are shown enlarged in Figure 4. Here, most of the aggregates are flexible worms, i.e., chainlike aggregates with a small bending modulus. The geometry of the worm backbone is dictated by the necessity to keep the energy as small as possible at the given composition: this was accomplished by a straight chain of disks for χ=33% (Figure 4, left panel) and by a zig-zag chain for χ=50% (Figure 4, right panel). Both chain morphologies allowed for disks to bind all dimers, so that, in the long run, no free particles would be left in the box. Occasionally, a worm bent to the point that a closed loop appears—see the example on the left in Figure 4.

**Figure 5:**
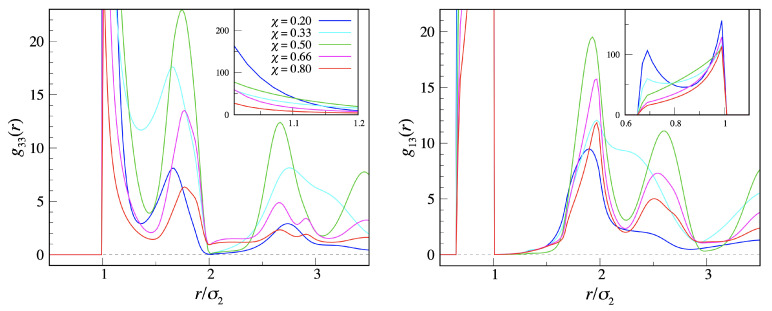
Mixture of dimers and disks with $\sigma_{3}=\sigma_{2}$ after $10^{8}$ MC cycles for $T^{*}=0.10$ and $\rho^{*}=0.05$. (**left**) $g_{33}\left(r\right)$; (**right**) $g_{13}\left(r\right)$; (inset) short-range structure of RDFs. The color code is the same for both panels (see left-panel inset).

Figure 5 shows the collected RDFs for various compositions at low temperature ($T^{*}=0.10$). A large $g_{33}$ value at contact is the most distinct signature of the existence of aggregates of disks. The short-distance structure in $g_{33}$ was richer for intermediate χ values, where the physiognomy of aggregates is better defined. The rather pronounced second-neighbor peak at χ=50% was the result of the zig-zag structure of the chain backbone at this composition. Regarding $g_{13}\left(r\right)$, its short-distance profile was sharper for the lowest compositions, where the number of dimers that were in close contact with the same sphere was higher (see inset of right panel). A high third-neighbor peak for intermediate compositions is the signature of the existence of extended aggregates of dimers and guest disks consisting of a periodically repeating unit.

**Figure 6:**
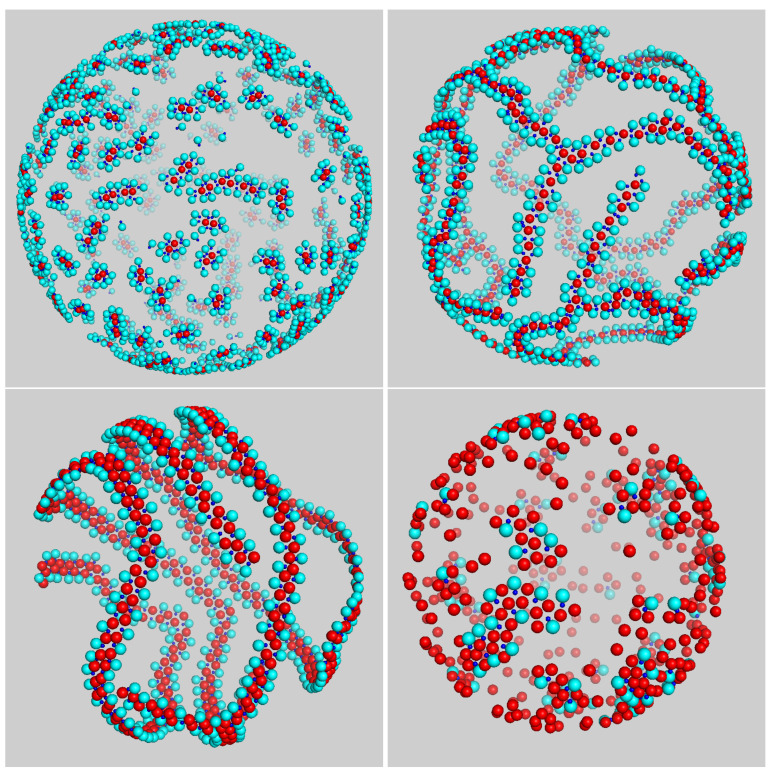
Typical configuration of mixture for $\sigma_{3}=\sigma_{2}$ and $\rho^{*}=0.25$ after a long run carried out at $T^{*}=0.10$. Snapshots were taken at different compositions: (from **top left** to **bottom right**) χ=20%,33%,50%, and χ=80%.

As density increases, the nature of self-assembly becomes slightly different. We studied mixtures for $\rho^{*}=0.25$ while still keeping $\sigma_{3}=\sigma_{2}$ and the temperature fixed at 0.10 (see Figure 6). When the composition was low, the aggregates were slightly elongated capsules (see top-left panel of Figure 3), like at small density. For χ=33%, aggregates were definitely chainlike (Figure 6, top-right panel), but due to a more crowded environment, they were joined together in an intricate manner, giving rise to a spanning cluster (i.e., a connected gel-like network encompassing all particles in the system). The onset of an extended network is a remarkable outcome, considering that this structure emerged from very basic interaction rules in a binary system of disks and dimers. At the composition of 50%, large monomers were more effective in screening a chain from other aggregates, and chains then grew much longer. As a result, a chain may wrap a few times around the sphere before merging into another chain (see bottom-left panel of Figure 6). This is similar to what was observed in a one-component system of particles interacting through a short-range attractive, long-range repulsive (SALR) potential [35]. The latter system was stripe-forming at low temperature; hence, when the particles were constrained to a spherical surface, a stripe may wrap around the sphere, as indeed observed. Lastly, at still higher compositions of disks, the size of aggregates was again reduced since there were few disks to bind all dimers, and their environment was too crowded to allow for aggregates to grow long in the early stages of equilibration.

While a further, moderate increase in density at fixed N≈1000 would not change much in the above results, in a huge-density mixture, the formation of self-organized aggregates could encounter much difficulty due to increased relaxation time and surface overcrowding.

### 3.2. Increasing Disk Size

**Figure 7:**
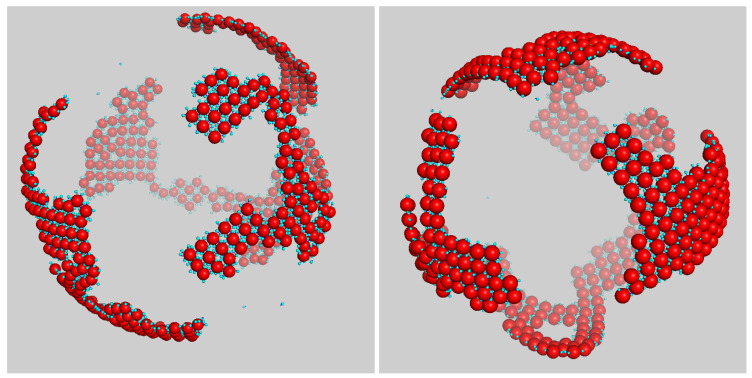
Typical configuration of mixture for (**left**) $\sigma_{3}/\sigma_{2}=4$ and (**right**) $\sigma_{3}/\sigma_{2}=5$ after a long run at $T^{*}=0.10$. System density was $\rho^{*}=0.05$ and disk composition was χ=20%. Disks were gathered together in patches similar to tectonic plates floating on Earth’s mantle. Prevailing structural motif was a square of disks with gluing dimers in the middle interstice.

When the size of disks became sufficiently large, four at least, the large monomers failed to adequately screen the attraction between two disk aggregates, and, at low temperature, we observed the formation of a condensate over the sphere in agreement with what was found for the system absorbed on a flat interface [31]. This is clearly seen in the snapshots reported in Figure 7, which correspond to a typical late-time configuration of the system for $T^{*}=0.10,\rho^{*}=0.05$, and χ=20%. For $\sigma_{3}/\sigma_{2}=5$, disks mostly occurred in the form of a square-symmetric polycrystalline structure, held together by dimers interspersed between the disks (we counted an average of four dimers in each square center, in accordance with the overall disk composition). The prevalent square order of the system was transparent in the profile of $g_{33}\left(r\right)$, where the second and third peaks fall at distances that are in a ratio of $\sqrt{2}$ and 2, respectively, with the location of the first peak. Figure 7 evidently shows that the size of patches with a clear square motif was nonetheless limited, and the reason for this is twofold. First, this may have been the result of an incomplete equilibration; relaxation to equilibrium (coarsening) is slower for a crystallizing system in which many particles are hosted in a single cell. As a result, the spontaneous elimination of crystalline defects takes much longer than it would in a one-component crystal of spherical particles. Even though the onset of crystalline order is favored on a sphere by the lowering of the nucleation barrier [36], this effect was seemingly small at the probed densities where the curvature of the sphere was also small. On the other hand, perfect square order is inherently frustrated on the sphere, and this placed an upper threshold on the size of the ordered patches (this would still be consistent with the existence of a superstructure of patches, akin to the icosahedral superstructure found in dense systems of hard disks on a sphere [37], but we have no evidence for that).

The regular structure observed in Figure 7 finds a correspondence in the crystalline order of DNA-hybridized polystyrene colloids on the surface of oil droplets, as shown in the fluorescence micrographs of [25]. At variance with these triangular-ordered crystalline patches, however, our system is unique in providing a square-symmetric scaffold for the absorption of foreign particles on a spherical substrate.

## 4. Conclusions

We performed Monte Carlo (MC) computer simulations on a sphere of a binary mixture of asymmetric dimers of tangent hard disks and guest particles at low temperature. Guest particles were curved hard disks interacting with the smaller monomer of the dimer through an attractive square-well potential, of which the range was the same as the monomer diameter. We analyzed the effect of changing the density of the mixture (while keeping the system very sparse), the composition, and the size of guest particles, ranging from the size of the larger monomer in a dimer to five times larger than that. Despite the simplicity of the model, its self-assembly behavior was quite rich. Indeed, we observed the formation of various metastable aggregates with a prevailing one-dimensional geometry (i.e., chainlike aggregates with a small bending modulus, including a gel-like network), only driven by the short-range attraction, while the large-scale distribution of aggregates could be understood in terms of entropic considerations. For a large guest particle, the small monomer–guest particle attraction could only hardly be screened by the large monomer, thus favoring the formation of thick condensates. These condensates appeared as a square-symmetric polycrystal, which is a nontrivial feature of the model, considering that the perfect square lattice is inherently frustrated on the sphere. Our results indicate the possibility of building a square-symmetric scaffold for the absorption of external particles on a curved surface.

## Data Availability

Data are available on request from the corresponding author.
